# 8-year-old boy with reduced vision in both eyes

**DOI:** 10.4103/0974-620X.53044

**Published:** 2009

**Authors:** George J. Manayath, Rana Al-Senawi

**Affiliations:** Department of Ophthalmology, Sultan Qaboos University Hospital, Muscat, Sultanate of Oman

## Questions:

An 8-year-old boy presented with reduced vision in both eyes (OU) since six years.

1. What is the diagnosis? Describe two features suggestive of the diagnosis.
2. What are the main causes of visual loss in this condition?

## Answers

**Figure 1:**
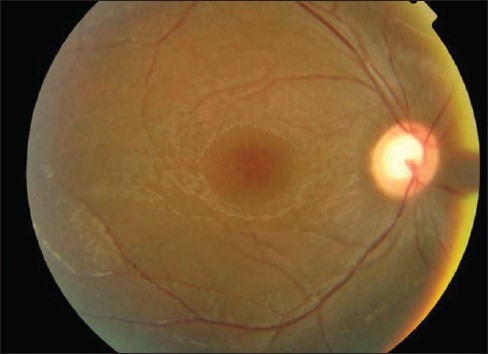
Fundus photograph right eye (OD)

**Figure 2:**
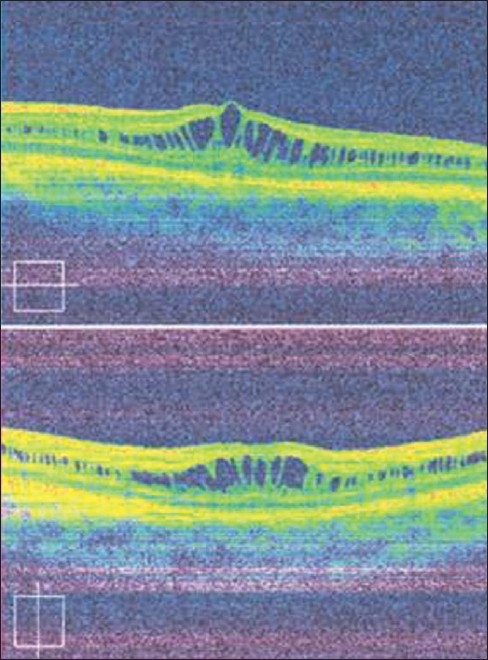
Optical coherence tomography (OCT) OD

**Diagnosis:** X-linked Retinoschisis. Two features suggestive of diagnosis: (1) Retinal folds radiating from the center of fovea (petalloid pattern) suggestive of foveal schisis or ′bicycle wheel′ maculopathy [Figure 1]. (2) Optical coherence tomography (OCT) image shows multi-layered schisis cavities bridged by vertical hyper-reflective columns [Figure 2].

**Causes of Visual Loss:** Maculopathy, vitreous hemorrhage and retinal detachment.

## Comments

### X-linked Retinoschisis

Congenital retinoschisis is a rare vitreoretinal degeneration that occurs due to splitting of nerve fiber layer of retina, and has an X-linked recessive inheritance; thus mainly affecting males. Central vision may initially be good with reading difficulties developing in the first decade due to maculopathy, but may drop with time typically to 20/200 due to macular degeneration. A constant diagnostic feature in pediatric patients is foveal schisis or ′bicycle wheel′ maculopathy, which appears as small, superficial cystoid spaces and retinal folds radiating from the center of fovea (petalloid pattern). Typically, fluorescein angiography shows no leakage. The classic pattern on OCT image is multi-layered schisis cavities, bridged by vertical hyper-reflective columns. Peripheral schisis is seen in 50% cases, typically involving the inferotemporal quadrant and is bilateral in 40% cases. The inner layer may develop oval holes and shows pigmented demarcation lines even when retina is not detached. Electroretinography gives an attenuated b-wave "negative waveform" with normal a-wave, suggesting panretinal dysfunction. Dark adaptation is normal or minimally affected. The two main complications which lead to severe visual deterioration are vitreous hemorrhage and retinal detachment.

